# Recommendations for mRNA analysis of micro-dissected glomerular tufts from paraffin-embedded human kidney biopsy samples

**DOI:** 10.1186/s12867-018-0103-x

**Published:** 2018-03-13

**Authors:** Clemens L. Bockmeyer, Juliane Wittig, Karen Säuberlich, Philipp Selhausen, Marc Eßer, Philip Zeuschner, Friedrich Modde, Kerstin Amann, Christoph Daniel

**Affiliations:** 1 Department of Nephropathology, Institute of Pathology, Friedrich-Alexander University Erlangen-Nuremberg, Krankenhausstraße 8-10, 91054 Erlangen, Germany; 2 Institute of Pathology, Hannover Medical School, Hannover, Germany; 3 Institute of Pathology, University Hospital of Cologne, Cologne, Germany

**Keywords:** Laser capture micro-dissection, LCM, Laser microdissection, Kidney biopsies, FFPE, Endogenous controls, Reference transcript, Housekeeping gene

## Abstract

**Background:**

Glomeruli are excellent pre-determined natural structures for laser micro-dissection. Compartment-specific glomerular gene expression analysis of formalin-fixed paraffin-embedded renal biopsies could improve research applications. The major challenge for such studies is to obtain good-quality RNA from small amounts of starting material, as applicable for the analysis of glomerular compartments. In this work, we provide data and recommendations for an optimized workflow of glomerular mRNA analysis.

**Results:**

With a proper resolution of the camera and screen provided by the next generation of micro-dissection systems, we are able to separate parietal epithelial cells from glomerular tufts. Selected compartment-specific transcripts (WT1 and GLEPP1 for glomerular tuft as well as PAX2 for parietal epithelial cells) seem to be reliable discriminators for these micro-dissected glomerular substructures. Using the phenol–chloroform extraction and hemalaun-stained sections (2 µm), high amounts of Bowman’s capsule transections (> 300) reveal sufficient RNA concentrations (> 300 ng mRNA) for further analysis. For comparison, in unstained sections from a number of 60 glomerular transections upwards, a minimum amount of 157 ng mRNA with a reasonable mRNA purity [A260/A280 ratio of 1.5 (1.4/1.7) median (25th/75th percentiles)] was reversely transcribed into cDNA. Comparing the effect of input RNA (20, 60, 150 and 300 micro-dissected glomerular transections), transcript expression of POLR2A significantly correlated when 60 and 150 laser micro-dissected glomerular transections were used for analysis. There was a lower inter-assay coefficient of variability for ADAMTS13, when at least 60 glomerular transections were used. According to the algorithms of geNormPlus and NormFinder, PGK1 and PPIA are more stable glomerular reference transcripts compared to GUSB, GAPDH, POLR2A, RPLPO, TBP, B2M, ACTB, 18SrRNA and HMBS.

**Conclusions:**

Our approach implements compartment-specific glomerular mRNA expression analysis into research applications, even regarding glomerular substructures like parietal epithelial cells. We recommend using of at least 60 micro-dissected unstained glomerular or 300 hemalaun-stained Bowman’s capsule transections to obtain sufficient input mRNA for reproducible results. Hereby, the range of RNA concentrations in 60 micro-dissected glomeruli is low and appropriate normalization of C_q_ values using our suggested reference transcripts (PGK1 and PPIA) allows compensation with respect to different amounts of RNA purity and quantity.

**Electronic supplementary material:**

The online version of this article (10.1186/s12867-018-0103-x) contains supplementary material, which is available to authorized users.

## Background

Laser capture micro-dissection (LCM) enables the isolation of single cells or small cell groups from histological sections under direct microscopic control [1]. Combined with quantitative PCR, LCM is a very powerful approach for studying individual transcript expression profiles in discrete cell populations [2]. Especially for renal biopsies, micro-dissection of its different compartments—glomeruli, tubulointerstitium and arteries/arterioles—is a worthwhile exercise despite its small amounts of degraded RNA. About 17 years ago, glomerular micro-dissection was established in frozen sections obtained from a rat model [3]. The method itself [4–6] and its potential role in diagnostic work-up of renal biopsies [7, 8] were reviewed several years ago. Hereby, general remarks for the major challenge for such approaches to obtain RNA with sufficient quantity and proper quality from small amounts of starting material are given, but no data for experimental procedures in detail. Especially, it has been shown that the majority of studies were performed with frozen tissue. This limited experience with formalin-fixed paraffin-embedded (FFPE) studies is due to the fact that frozen tissue is preferred, which is most often available from animal studies. However, often this is hardly an option for the large-scale analysis of human FFPE tissue from renal biopsies.

A comprehensive study for RNA quality and quantity was recent performed for the first time in renal FFPE tissue [9]. Furthermore, six studies [9–13] including ours [14] have performed successful mRNA gene expression analysis from micro-dissected FFPE glomerular transections using different methodological approaches. Another study described a selection of reference transcripts for mRNA quantification from these specimen [15]. However, there was no clear-cut recommendation for glomerular reference mRNA transcripts and renal biopsies stored in RNAlater [15]. Most often, GAPDH (glyceraldehyde-3-phosphate dehydrogenase) is recommended as a glomerular reference transcript, although there is still no systematic study upon which the preference for GAPDH can be based [16].

Therefore, one aim of this study was to identify stably-expressed reference transcripts by proper normalization strategies and compare our approach for quantitative mRNA analysis of micro-dissected glomerular compartments with others. Another focus was to illustrate the possibilities for laser micro-dissection of glomerular substructures in detail. In addition, we studied the effect of the amount of micro-dissected glomerular transections for further RNA purity and quantity control, especially the net result for relative quantification. Hereby, we focused on two candidate reference transcripts (GAPDH and POLR2A [polymerase (RNA) II (DNA directed) polypeptide A)] and one target transcript [ADAMTS13 (a disintegrin and metalloproteinase with thrombospondin type 1 motives 13)] due to our interest in thrombotic microangiopathy.

## Methods

### Patients and biopsies

Overall, FFPE native and transplant kidney biopsies from 24 patients were selected from the archive of the Institute of Pathology, Hannover Medical School and Institute of Pathology, Department of Nephropathology, Friedrich-Alexander University Erlangen-Nuremberg. (i) Nine native kidney biopsies with acute tubular injury (ATN) or interstitial inflammation without any glomerular disease were selected for the practicability of the micro-dissection of parietal epithelial cells (PECs, patient cohort I). (ii) In order to determine reference transcripts, we used an additional fifteen patients selected by a high number (> 30) of glomeruli per biopsy, including a wide range of native and transplant biopsies (patient cohort II). These patients had different glomerular, tubulo-interstitial and hypertension-related renal diseases. Eight of these patients were analyzed for the effect of input RNA. Patient characteristics for all biopsies are detailed in Table 1. Clinical data included blood pressure, proteinuria (none, non-nephrotic, nephrotic), hematuria and serum-creatinine. There were significantly more hypertensive patients in cohort II compared to cohort I (p = 0.0234; Mann–Whitney U test). We could not find any significant differences for other clinical data between different patient groups.

**Table 1:** Patient characteristics

	Diagnosis	Female	Age at biopsy	Hypertension	Hematuria	Proteinuria	Creatinine (mg/dl)
Cohort I							
	- ATN (9/9) - Mild interstitial inflammation (4/9) - Mild interstitial nephritis (5/9)	5/9	37 (20/63)	1/9	4/9	7/9	3.1 (1.6/5.3)
Cohort II							
	- Unspecific minimal structural changes - Acute tubular injury - Lupusnephritis class II - Hypertensive ischemic nephropathy - Diabetic nephropathy - Minimal change disease - Primary focal-segmental Glomerulosclerosis - Humoral rejection type II (n = 3) - Acute tubular injury after transplantation (n = 4) - Mild interstitial fibrosis after transplantation	7/15	50 (37/61)	9/15	3/13	6/13	1.5 (0.8/3.1)

Main data of the different cohorts are summarized: Cohort (I) for micro-dissection of glomerular substructures, cohort (II) for comparing different glomeruli counts and for reference transcript analysis. Cohort (I) comprised of nine patients with non-glomerular diseases, and cohort (II) of fifteen patients (seven native and eight transplant biopsies) randomly selected by a high number of glomeruli per biopsy. Two patients were on dialysis and no data for proteinuria and hematuria were available. There was a significant difference for hypertension between cohort I and II (p = 0.0234; Mann–Whitney U test). Data are given as median (25th/75th percentiles) or absolute values

### Micro-dissection and mRNA analysis

All biopsies were cut at a thickness of 2 µm. In order to test the practicability of micro-dissection of PECs (patient cohort I), biopsies were deparaffinized for 30 s in xylol, stained for 2 s in hemalaun (Mayers Hemalaun, Merck, Germany, Cataloge number 109249) and washed in sterile aqua ad injectabilia (Ampuwa, Plastipur, Fresenius, Germany, Cataloge number 1088813). After the drying of several consecutive sections, non-sclerotic glomerular tufts were micro-dissected from PECs using a next generation micro-dissection system (MMI^®^ CellCut Plus^®^ Laser-Micro-dissection System, Molecular Machines and Industries (MMI), Eching, Germany and MMI^®^ CellCamera DXA285cF) with the following settings: energy: 70%, focus: 50%, cutting speed: 40%.

Several non-deparaffinized and unstained consecutive transections were applied for the patient cohort II. Micro-dissection was performed by another micro-dissection system (Zeiss/Palm MicroBeam AxioVert 200M, Camera Hitachi HV-D30, Crystal Laser systems FTSS 355-50) with the same laser settings. For reference transcript analysis, 60 glomerular transections from all fifteen patient samples were used. In order to determine the effect of input RNA for valid mRNA expression analysis, 20, 60, 150 and 300 glomerular transections from eight patient samples out of cohort II were used.

RNA was quantified as previously described [17, 18]. Briefly, RNA was isolated by phenol–chloroform extraction and dissolved in 11 µl DEPC water. Subsequently, 1 µl was used for measuring the RNA amount and A260/A280 ratio by a spectrophotometer (Synergy, Biotek Instruments, Bad Friedrichshall, Germany). The remaining 10 µl RNA solution was reversely transcribed using the Multi-Scribe-based High Capacity Kit (ThermoFisher Scientific previously Applied Biosystems, Foster City, CA, USA). For quantitative mRNA analysis, we used reverse transcription single TaqMan real-time quantitative PCR (RT-qPCR) with TaqMan pre-designed assays after cDNA synthesis and preamplification (ThermoFisher Scientific). The TaqMan assay identity numbers and amplicon sizes of ADAMTS13 and selected candidate reference transcripts [PGK1 (Phosphoglyceratekinase 1), POLR2A, GAPDH, GUSB (Beta glucuronidase), PPIA (Peptidylprolyl isomerase A *Alias:* Cyclophilin A), ACTB (Beta-Actin), eukaryotic 18S rRNA, B2M (Beta-2-microglobulin), HPRT1 (Hypoxanthine phosphoribosyltransferase 1), RPLPO (ribosomal protein lateral stalk subunit P0), TBP (TATA-box binding protein) and HMBS (hydroxymethylbilane synthase)] as well as compartment markers WT1 (Wilms tumor 1) for podocytes/PECs, PAX2 (Paired box 2) for PECs/tubulointerstitium and GLEPP1 (*Alias:* PTPRO (Protein tyrosine phosphatase, receptor type O) for podocytes/PECs are detailed in Table 2. No-reverse transcription controls and no-template controls were included during each amplification step. Off-scale C_q_ values (C_q_ > 35) and missing C_q_ values were replaced by C_q_ = 36 [19].

**Table 2:** TaqMan assay identity numbers, amplicon size and characteristics of analyzed candidate reference and target transcripts

Gene symbol (full name)	TaqMan assay identity number	Amplicon size	Chromosomal location	Function
PGK1 (Phosphoglycerate kinase 1)	Hs00943178_g1	73	Xq21.1	Kinase that catalyzes the conversion of phosphoglycerates; also involved in tumor angiogenesis
POLR2A (RNA polymerase II subunit A)	Hs00172187_m1	61	17p13.1	Synthesizing mRNA
GAPDH (Glyceraldehyde-3-phosphate dehydrogenase)	Hs99999905_m1	122	12p13.31	Important in carbohydrate metabolism
GUSB (Glucuronidase, beta)	Hs99999908_m1	81	7q11.21	Hydrolases glycosaminoglycans
PPIA (Peptidylprolyl isomerase A)	Hs99999904_m1	98	7p13	Catalyzes the cis–trans isomerization of peptide bonds in oligopeptides. Possible role in cyclosporine A-mediated immunosuppression
ACTB (Beta-Aktin)	Hs01060665_g1	63	7p22.1	Nonmuscle cytoskeletal actin that is ubiquitously expressed
Eukaryotic 18S rRNA	Hs03003631_g1	69	12	Catalyzes the synthesis of peptides and proteins
B2M (Beta-2 microglobulin)	Hs00187842_m1	64	15q21.1	Protein found in association with the major histocompatibility complex (MHC) class I heavy chain on the surface of nearly all nucleated cells
HPRT1 (Hypoxanthine phosphoribosyltransferase 1)	Hs02800695_m1	82	Xq26.2-q26.3	Plays a central role in the generation of purine nucleotides
RPLPO (Ribosomal protein lateral stalk subunit P0)	Hs00420895_gH	76	12q24.23	Encodes a ribosomal protein that is a component of the 60S subunit
TBP (TATA-box binding protein)	Hs00427620_m1	91	6q27	Coordinates Initiation of transcription by RNA polymerase II
HMBS (Hydroxymethylbilane synthase)	Hs00609297_m1	64	11q23.3	Encodes a member of the hydroxymethylbilane synthase superfamily
WT1 (Wilms tumor 1)	Hs01103751_m1	72	11p13	Development of the urogenital system
PAX2 (Paired box 2)	Hs00240858_m1	57	10q24.31	target of tumor suppressor gene WT1
PTPRO (Protein tyrosine phosphatase, receptor type O, alias GLEPP1)	Hs00958189_m1	91	12p12.3	Regulation of osteoclast production and activity, inhibition of cell proliferation and facilitation of apoptosis
ADAMTS13 (A disintegrin and metalloproteinase with thrombospondin type 1 motives 13)	Hs00260148_m1	59	9q34.2	Cleaves von Willebrand factor

### Immunostaining

Immunohistochemical staining was performed exemplarily in cases with ATN and mild interstitial inflammation (patient cohort I). All staining procedures were undertaken manually with the ABC detection system (peroxidase) from Vector Laboratories PK-6100 (Burlingame, CA, USA). Treating sections with 3% hydrogen peroxide for 10 min quenched endogenous peroxidase activity. Antigen retrieval for PAX2, WT1 and GLEPP1 was performed with Tris buffer pH 6.0 for 2.5 min at 120 °C in a pressure cooker. Secondary antibody was incubated for 30 min at room temperature at a dilution of 1:200. Diaminobenzidine (DAB) was used for a brown reaction product at the site of bound primary antibody.

In detail, for PAX2 immunostaining a rabbit polyclonal antibody (71-600, ThermoFisher, Foster City, CA, USA) was used at a dilution of 1:500. WT1 immunostaining was conducted with overnight incubation (4 °C) with a rabbit polyclonal antibody (RB-9267, ThermoFisher, Foster City, CA, USA) at a dilution of 1:10,000. GLEPP1 immunostaining was conducted with a rabbit polyclonal antibody (HPA034525, Sigma-Aldrich, Munich, Germany) at a concentration of 1:5000 with overnight incubation (4 °C).

### Statistics

Non-parametric Wilcoxon matched-pairs signed rank test was used for comparison of all data except for comparison of clinical data between cohort I and II (Mann–Whitney U test). Median values as well as 25th/75th percentiles are shown in the box plots or given in the text. Furthermore, absolute data or Spearman rank correlations are shown. Graphical and statistical analyses were performed using GraphPad Prism Version 5.0 (GraphPad Software Inc., La Jolla, CA, USA).

The NormFinder and geNormPlus approach was used as recently described [20]. Briefly, NormFinder considers the intra- and inter-group variations independently. The stability value for each gene is a measurement of the estimated systematic error when using this gene for normalization. The commercially-available geNormPlus algorithm in qBase^plus^, version 3.0 (http://www.biogazelle.com/qbaseplus Biogazelle, Zwijnaarde, Belgium) is based on calculating the average pairwise variations of each candidate reference gene compared to all candidate reference genes [21]. For the identification of the optimal number of reference genes, geNormPlus provides the following algorithm: differences in the ratio of the average pairwise variation of normalization factors (geometric mean C_q_ value) [21] of consecutive candidate reference genes starting with the two most stable genes (n) and the addition of the next most stable gene (n + 1) until all genes have been added suggest the number of optimal reference genes. As a general guideline, the benefit of using an additional reference gene is limited as soon as the difference in the ratio (Vn/n + 1) drops below the 0.15 threshold [22].

According to our results of NormFinder and geNormPlus, we used the geometric mean of PGK1 and PPIA for normalization of glomerular ADAMTS13, GAPDH and POLR2A transcript expression. By contrast, for comparison of expression levels in PEC, tubulointerstitium and glomeruli we did not perform a NormFinder and geNormPlus analysis. Therefore, the geometric mean of GAPDH and PGK1 was used for normalization of cohort I. This decision was based on the fact, that GAPDH has been used for normalization in tubulointerstitial studies [23, 24].

## Results

### Visualization of hemalaun-stained glomerular substructures and subsequent compartment-specific transcript analysis

**Fig. 1:**
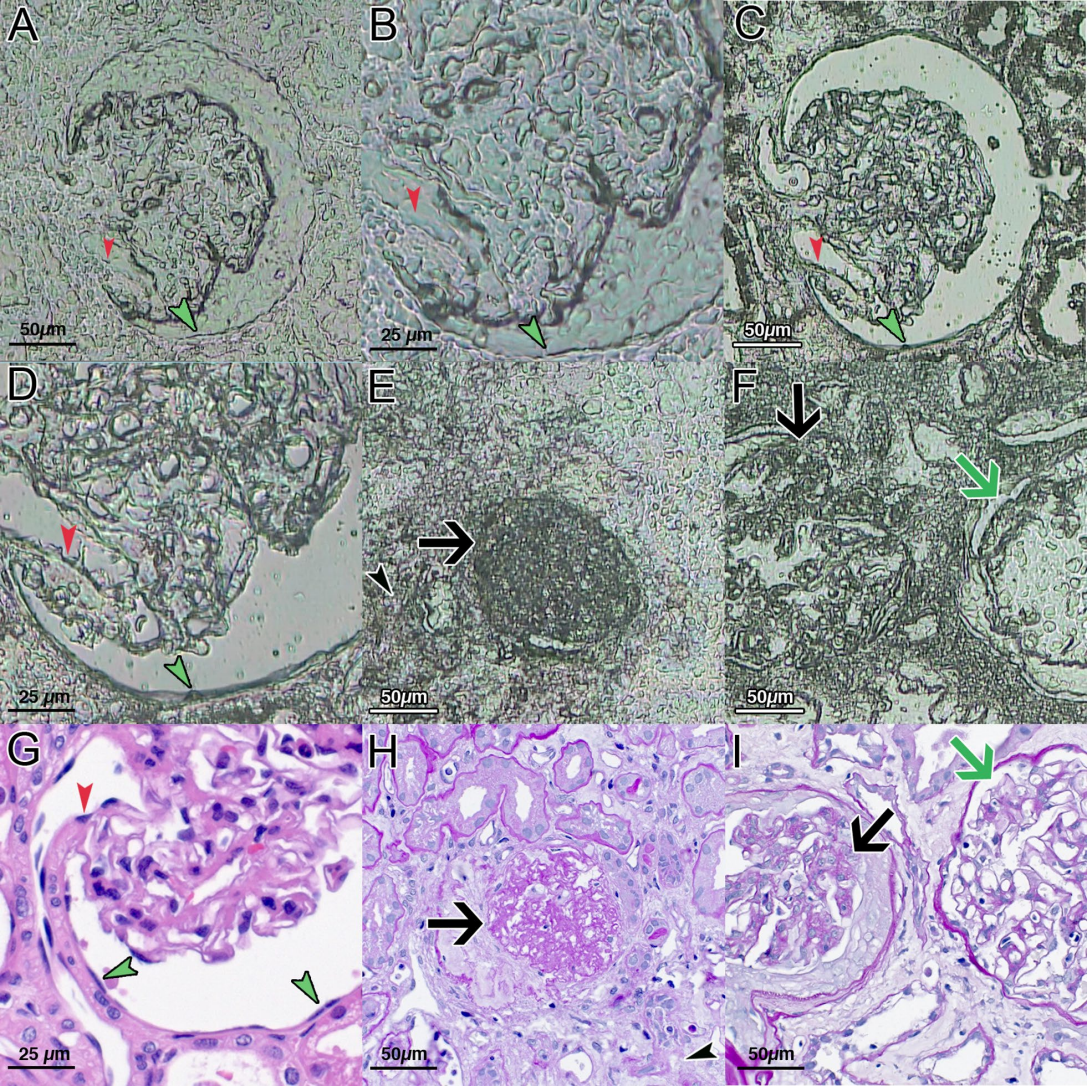
Different resolution of defined glomerular structures in dependence on deparaffinization. Without deparaffinization (**A**, **B**), only coarse structures are visible. However, deparaffinization and a short hemalaun stain allow the detection of nuclei of PECs (green arrowheads) and endothelial (red arrowheads) cells (**C**, **D**). By contrast, globally (**E**, black arrow) or segmentally (**F**, black arrow) sclerosed glomeruli as well as scarred tubulo interstitium (**E**, black arrowhead) can easily be detected without deparaffinization. One glomerulus with open capillary loops is shown in comparison (**F**, green arrow). HE (**G**) and PAS (**H**, **I**) stains from consecutive sections of **D–F** are given. PALM/Zeiss laser technique and software, magnification ×400 or ×200, **C**, **D** short hemalaun stain

One main goal was to push the visual resolution of glomerular structures to the limits, especially to prove the reliability of micro-dissection for the isolation of PECs. First, we compared non-deparaffinization (Fig. 1A, B) with deparaffinization by xylol followed by hemalaun staining (Fig. 1C, D). Non-deparaffinization is an excellent option for the micro-dissection of glomerular capillaries and to identify sclerosed glomeruli (Fig. 1E, F) or scarred tubulointerstitium. There was no difference in visualization comparing 2 and 3 µm sections, although non-deparaffinized 2 µm sections were much easier to dissect with the laser beam. Without deparaffinization (Fig. 1A, B), it was virtually impossible to differentiate PECs. However, following deparaffinization and a short hemalaun stain, it was feasible to detect nuclei of PECs and endothelial cells (Fig. 1C, D). For comparison, HE (Fig. 1G) or PAS (Fig. 1H, I) stains are given for the samples above (Fig. 1D–F).

**Fig. 2:**
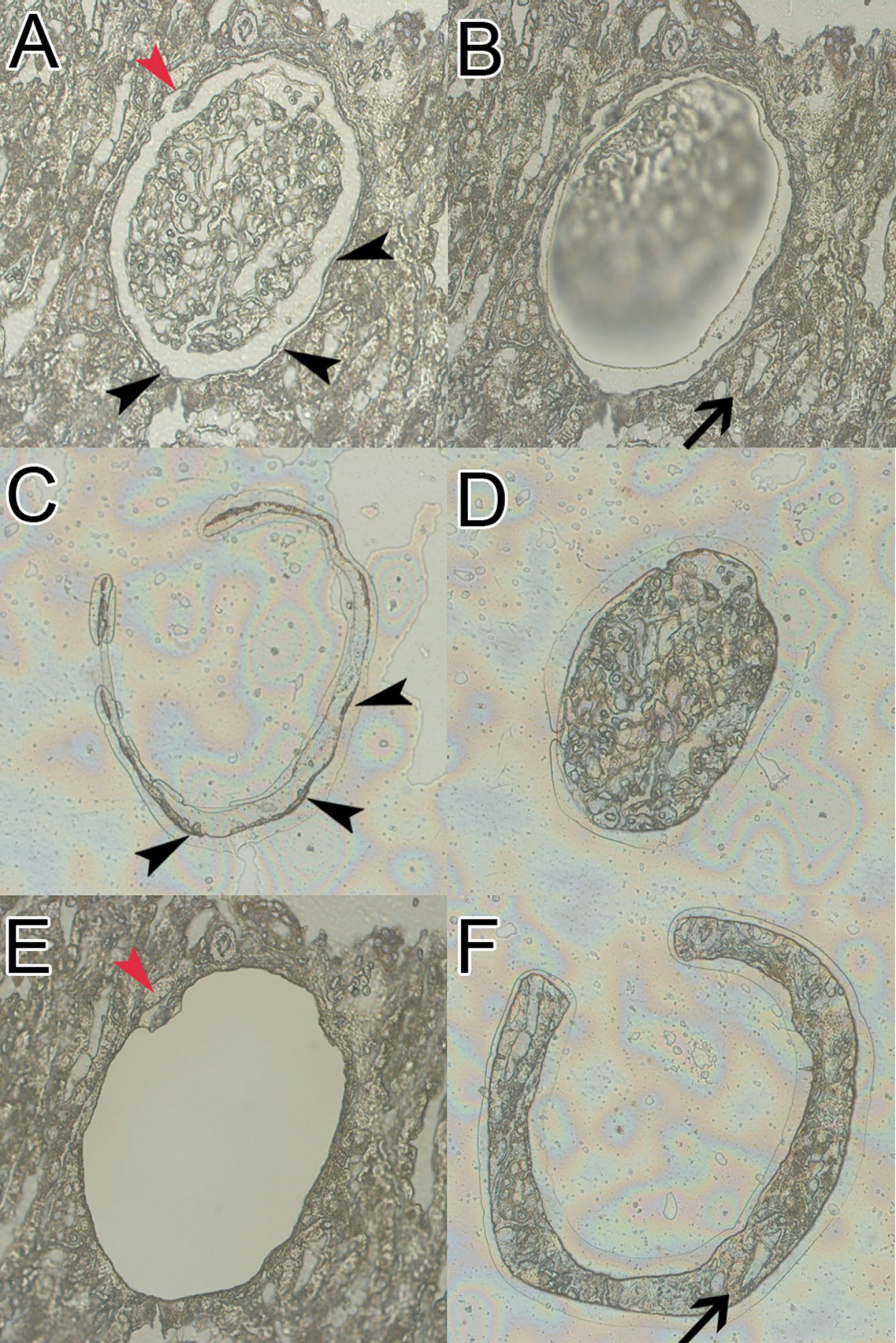
Advanced technique for micro-dissection of glomerular substructures. For this purpose, we used only deparaffinized sections followed by a short hemalaun stain to detect nuclei most effective and obtain a faint satisfying pattern. We micro-dissected three different compartments from paraffin-embedded human renal biopsies: glomeruli, parietal epithelial cells (PECs) and periglomerular tubulointerstitium. One exemplary glomerulus is shown (**A**). After micro-dissection of the glomerular tuft (**B**), PECs can be micro-dissected (black arrowheads, **C**) and are separated from the glomerular tuft (**D**) as well as discontinuous regions (**A**, **E**, red arrowhead). In the last step, periglomerular tubulointerstitium was micro-dissected (**F**). MMI laser technique and software, magnification ×400, short hemalaun stain

**Fig. 3:**
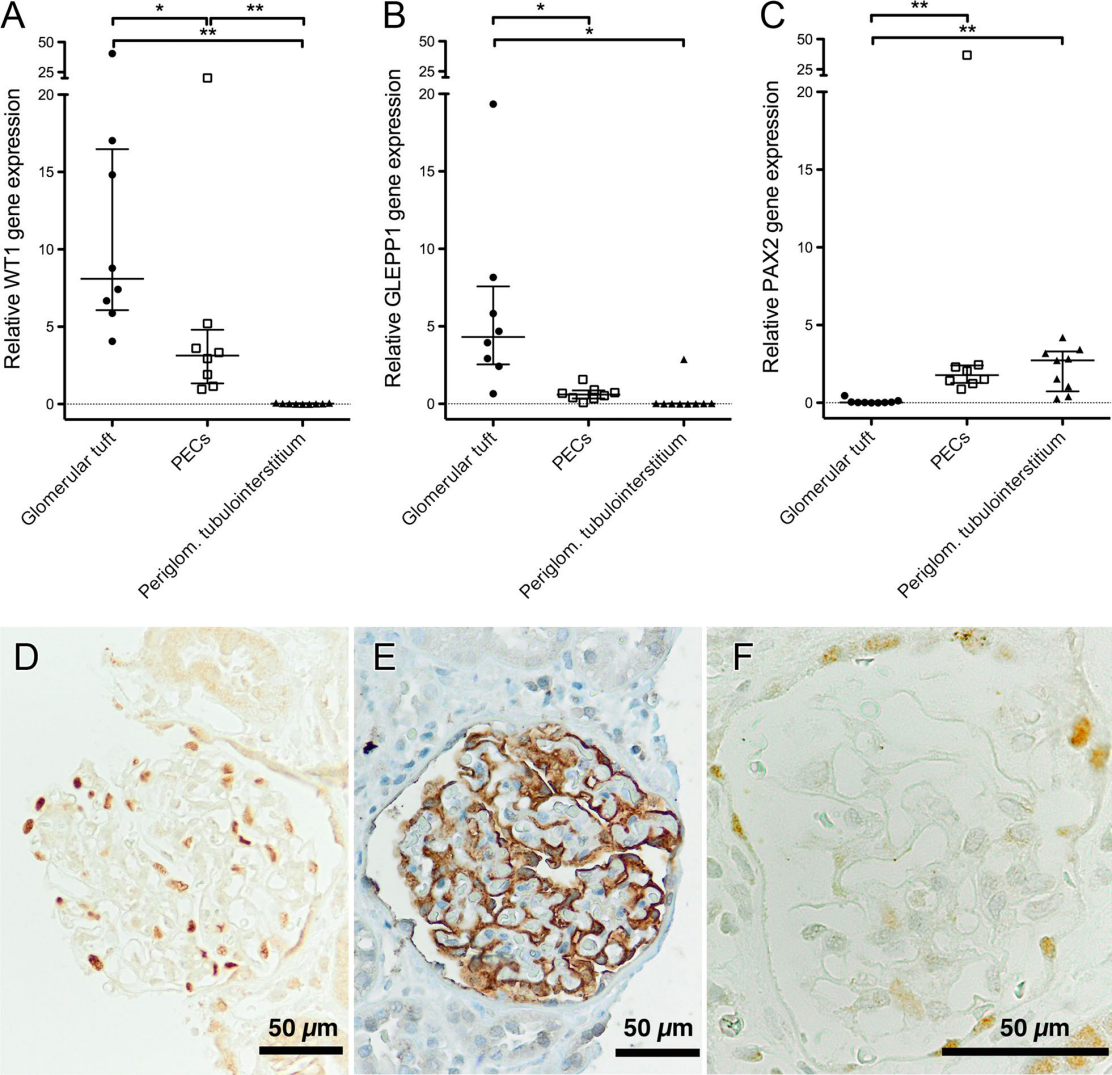
Expression levels of selected transcripts in micro-dissected non-injured hemalaun-stained glomerular substructures. Nine cases with acute tubular injury and mild interstitial inflammation, but without any glomerular disease were used (cohort I). WT1 (**A**), GLEPP1 (**B**) and PAX2 (**C**) seem to be reliable discriminators for proper micro-dissection of glomerular tufts vs. PECs. For comparison, periglomerular tubulointerstitium is shown. There were significant differences between glomerular tufts and PECs regarding all markers. Relative expression was calculated using the geometric mean of PGK1 and GAPDH as normalization factor. There was no expression of WT1 and GLEPP1 (beside one outlier) in periglomerular tubulointerstitium. In one patient case, no amplification of target and reference transcripts was detectable in PECs. One outlier was excluded in WT1 and GLEPP1 transcript expression in glomerular tufts. p-values were calculated by the Wilcoxon matched-pairs signed rank test (*p < 0.05; **p < 0.01). For comparison, immunostaining of WT1 (**D**), GLEPP1 (**E**) and PAX2 (**F**) is given in one selected case. WT1 (**D**) and GLEPP1 (**E**) are strongly expressed in podocytes and to a lower extend also in PECs, but not in the tubulointerstitium. PAX2 is expressed in PECs and tubular epithelial cells (**F**). Magnification ×400 or ×200

Moreover, the high-resolution camera and touch screen from the MMI system enabled micro-dissecting PECs, sparing the deparaffinized and hemalaun-stained glomerular tuft, especially in cases with a wide Bowman’s space (Fig. 2). For further characterization of micro-dissected non-injured glomerular substructures, we used a set of selected mRNA transcripts: WT1 (podocytes), GLEPP1 (podocytes) and PAX2 (PECs). Significantly different expression levels of WT1, GLEPP1 and PAX2 indicated an appropriate separation of substructures (Fig. 3A–C). The geometric mean of PGK1 and GAPDH was used as normalization factor for relative expression analysis of WT1, GLEPP1 and PAX2. For clarification of mRNA expression levels, immunostaining of WT1, GLEPP1 and PAX2 is provided. Both, WT1 (Fig. 3A, D) and GLEPP1 (Fig. 3B, E) were strongly expressed in podocytes and to a lower extend also in PECs, but not in the tubulointerstitium. PAX2 was expressed in PECs and tubular epithelial cells (Fig. 3C, F).

**Fig. 4:**
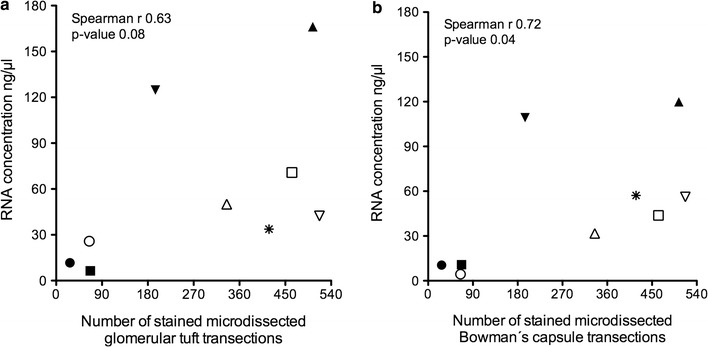
RNA concentrations in hemalaun-stained micro-dissected glomerular substructures. As expected, the RNA concentration tends to increase in dependence of the number of micro-dissected glomerular tufts (**a**) and Bowman’s capsule (**b**) transections. However, obtained amounts of RNA were not proportional to the number of glomerular transections. Interestingly, there is only a slightly higher RNA concentration in the glomerular tuft compared to Bowman’s capsule transections. Based on these data with a high fluctuation of RNA concentration in hemalaun-stained samples, we can only provide a conservative recommendation of using 300 *deparaffinized and stained* Bowman’s capsule transections, which reveal sufficient RNA concentrations of more than 30 ng/µl. Each symbol (open and filled triangles, squares, circles and one asterisk) represents one patient sample (n = 9; cohort I)

RNA quantities from these hemalaun-stained glomerular tufts and micro-dissected PECs in association with the number of micro-dissected glomerular transections are shown in Fig. 4. Interestingly, there were no significant differences in RNA concentration between glomerular tufts [42.4 (18.8/97.8) ng/µl]; Fig. 4a) and Bowman’s capsules [43.8 (10.7/83.3) ng/µl]; Fig. 4b) in case of 335 (66/484) micro-dissected glomerular transections. Among patients with less micro-dissected glomerular transections (about 60 or less), RNA concentrations below 10 ng/µl in PECs and a broad range between 6 and 26 ng/µl in glomerular tufts were found. There was also one sample with no detectable amplification signal in reference and target transcripts in case of 65 micro-dissected Bowman’s capsule transections. However, high amounts of Bowman’s capsule transections (> 300) revealed sufficient RNA concentrations (> 30 ng/µl; Fig. 4b).

### Reference transcripts for unstained glomerular transections

**Fig. 5:**
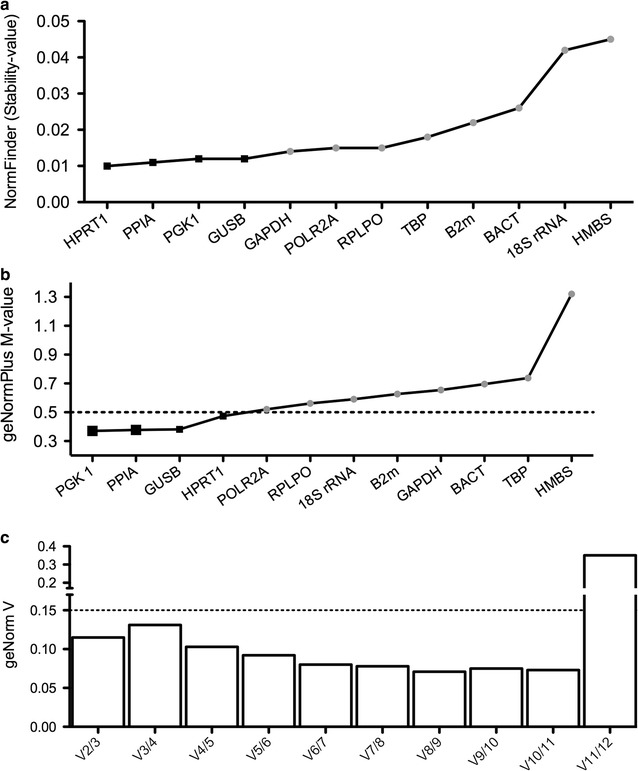
Stability of selected reference transcripts according to the algorithms of geNormPlus and NormFinder. Stability analysis was performed in cohort II (n = 15). The x-axis presents the ranking of reference transcripts in order of increasing stability from left to right. Transcripts with the highest stability value exhibit the least stable expression level, while those with the lowest stability values are the most stable, respectively. NormFinder stability values are listed in ascending order (**a**). The most stable (top four) transcripts are highlighted by black squares: HPRT1, PPIA, PGK1 and GUSB. geNormPlus analysis shows the calculation of the average expression stability M-value of selected reference transcripts determined by RT-qPCR. High stability is defined by an M-value of < 0.5 as indicated by the dotted line (**b**). PGK1, PPIA, GUSB and HPRT1 displayed a M-value < 0.5 (black squares). In addition, geNormPlus calculates the optimal number of reference genes, taking into account the variable V as the average pairwise variation between two sequential candidate reference transcripts (**c**). The ratio V of the best two (V2) and best three (V3) was below 0.15, indicating that the best two reference genes [PGK1 and PPIA; enlarged black squares (**b**)] are sufficient

Two established algorithms (NormFinder and geNormPlus) were used to define stable glomerular reference transcripts in micro-dissected glomerular transections from fifteen unstained samples. We included twelve different mRNA candidate reference transcripts (Fig. 5 and Table 2). In one sample, HMBS was not detected and thus this sample was excluded from further stability analysis. Both, NormFinder (Fig. 5a) and geNormPlus (Fig. 5b) analysis recommended PGK1, PPIA, HPRT and GUSB as the top four glomerular reference transcripts (black squares). However, depending on the algorithm, the order of stability of these four reference transcripts varied. According to geNormPlus, the optimal number of reference transcripts was two (Fig. 5c), indicated by the ratio of the average pairwise variation (V) of the best two (V2) and best three (V3) reference transcripts, which was below 0.15.

For the comparison of different studies using human FFPE renal biopsies for glomerular micro-dissection, we performed a literature search in PubMed looking for articles published until March 2017. There are five studies using different reference transcripts or genes (GAPDH, ACTB and Eukaryotic 18S rRNA) [9–13]. Detailed differences between these studies and our study are highlighted in Table 3. GAPDH, ACTB and Eukaryotic 18S rRNA were not recommended as reference transcripts in our analysis of unstained glomerular transections.

**Table 3:** Summary of studies using formalin-fixed human renal biopsies for glomerular micro-dissection and gene expression analysis of glomerular mRNA

First author	Sections cut in (µm)	Staining method	Number of micro-dissected glomerular transections	RNA extraction	RNA purity (A260/A280 ratio)	Total RNA quantity used for cDNA synthesis	Preamplification	Glomerular reference gene(s)	Lit.
Cohen	5	Hemalaun	20–50	Phenol–chloroform	nd	nd	None	GAPDH	[10]
Fries	5	None	10–25	Purescript RNA isolation kit (Gentra, Minneapolis, MN)	nd	nd	None	ACTB	[12]
Schmid	5	Hemalaun	20–50	RNeasy-Mini	nd	nd	None	GAPDH and 18 S rRNA	[11]
Landolt	10	HE	100	Various were compared	1.1–1.9	54–138 ng	Only pre-analytical steps were performed		[9]
Bao	Not given	None	50	mirVana miRNA Isolation Kit	nd	nd	None	ACTB	[13]
Bockmeyer	2	Comparing no staining and Hemalaun	Comparing 20–300	Phenol–chloroform	0.9 to 2.3	157–282 ng	Yes	PGK1, PPIA	This study

*nd* not determined

### mRNA expression in dependence of different amounts of unstained micro-dissected glomerular transections

**Fig. 6:**
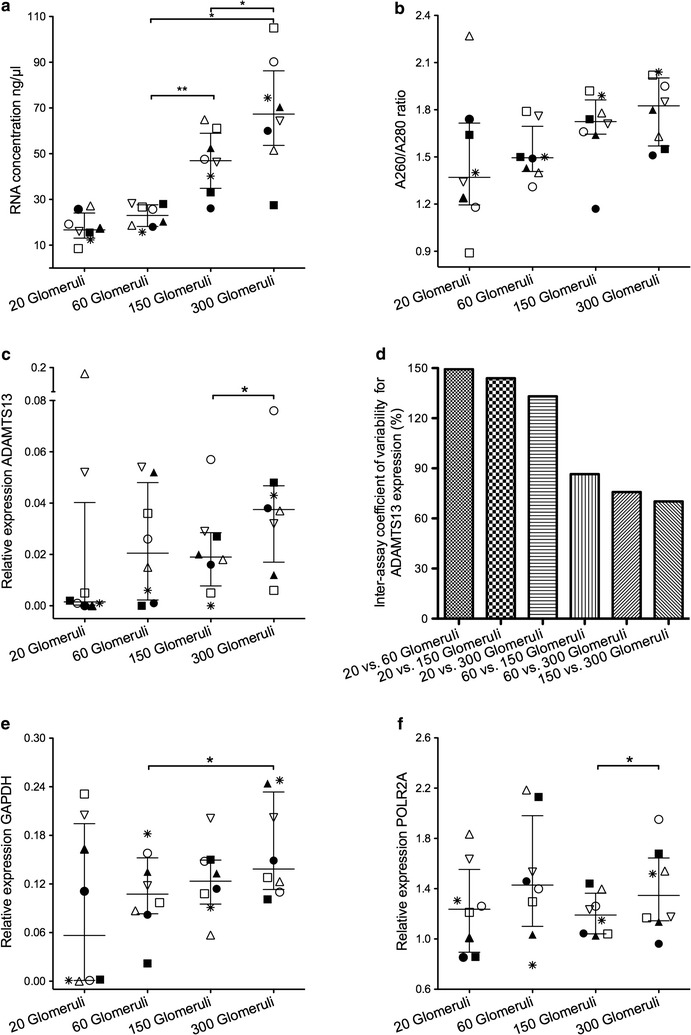
Comparing different unstained glomerular transection counts in eight cases of cohort II. Each case is depicted by one symbol (open and filled triangles, squares, circles, one asterisk and one upside down open triangle) in (**a**), (**b**), (**c**), (**e**) and (**f**). There was a significant difference in RNA concentration (**a**) comparing 60 and 150 as well as 150 and 300 unstained glomerular transections (termed as “glomeruli” for the sake of convenience; *p < 0.05; **p < 0.01; Wilcoxon matched-pairs signed rank test). There was no significant difference in the RNA A260/A280 ratio (**b**). Relative expression of ADAMTS13 (**c**) demonstrated a significant difference between 150 and 300 micro-dissected glomerular transections. The replacement of five C_q_ values of ADAMTS13 in 20 micro-dissected glomerular transections by C_q_ = 36 resulted in a relative expression of < 0.01 in those five cases (**c**). In groups with at least 60 glomerular transections the relative expression of ADAMTS13 displayed a lower inter-assay coefficient of variability compared to groups with 20 glomerular transections (**d**). The lowest inter-assay coefficient of variability for relative expression of ADAMTS13 was in groups with 150 and 300 glomerular transections (**d**). For comparison, relative expression of GAPDH and POLR2A is shown (**e**, **f**). In 150 micro-dissected glomerular transections, relative expression of ADAMTS13, GAPDH and POLR2A displayed the lowest interquartile range. There was a significant difference among groups with 60 and 300 glomerular transections for GAPDH (**e**) as well as 150 and 300 glomerular transections for POLR2A (**f**; *p < 0.05). Relative expression was calculated using PGK1 and PPIA as normalization factor

In order to determine the mRNA minimum required for the detection of reliable mRNA expression in unstained glomerular transections, we used ADAMTS13, GAPDH and POLR2A as target transcripts and the geometric mean of PGK1 and PPIA as normalization factor. To this end, we compared different amounts of micro-dissected glomerular transections: 20, 60, 150 and 300. The total amount of isolated RNA was between 16.7 (13.1/24.1) ng/µl for 20 micro-dissected glomerular transections and 67.4 (53.6/86.3) ng/µl for 300 micro-dissected glomerular transections (Fig. 6a). As expected, there was a significant increase in RNA concentration from 60 glomerular transections upwards (Fig. 6a). However, the amount of RNA was not proportional to the number of glomerular transections. With our protocol, we obtained mRNA with a purity of A260/A280 ratio between 0.9 and 2.3 from all samples (Fig. 6b). There were no significant differences regarding mRNA purity between different amounts of micro-dissected glomerular transections (Fig. 6b). Comparing the effect of input mRNA, in groups with 20 glomerular transections five C_q_ values of ADAMTS13 had to be replaced by C_q_ = 36 resulting in a relative expression of < 0.01 in those five cases. Relative expression of ADAMTS13 displayed a significant difference between 150 and 300 glomerular transections (Fig. 6c). In groups with at least 60 glomerular transections the relative expression of ADAMTS13 displayed a lower inter-assay coefficient of variability compared to groups with 20 glomerular transections (Fig. 6d). For comparison expression levels of GAPDH and POLR2A are given (Fig. 6e, f). There were no significant differences for all three transcripts ADAMTS13, GAPDH and POLR2A comparing expression levels between 60 and 150 glomerular transections. However, for 300 glomerular transections there was a significant difference compared either to 60 glomerular transections (GAPDH) or to 150 glomerular transections (POLR2A). The lowest interquartile range for ADAMTS13, GAPDH and POLR2A were in samples with 150 glomerular transections. Expression differences between ADAMTS13, GAPDH and POLR2A revealed similar significance levels, when 60 and 150 or 60 and 300 glomerular transection counts were compared (data not shown).

**Fig. 7:**
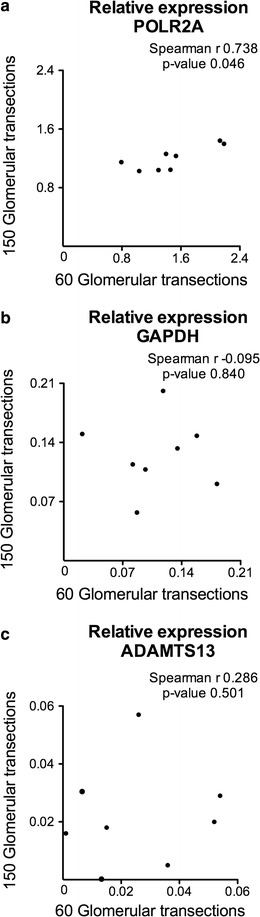
Comparing 60 and 150 unstained glomerular transections for relative expression in eight cases of cohort II. Relative expression levels of POLR2A (**a**), GAPDH (**b**) and ADAMTS13 (**c**) are shown normalized to PGK1 and PPIA. There was only a significant correlation for POLR2A expression (**a**). We could not find significant correlations regarding the gene expression of GAPDH and ADAMTS13 (**b**, **c**)

Furthermore, we tested the reproducibility of mRNA transcript levels between different amounts of micro-dissected glomerular transections. A minimum of 60 micro-dissected glomerular transections revealed a significant correlation for POLR2A expression compared to 150 micro-dissected glomerular transections (Fig. 7a) as well as between 150 and 300 micro-dissected glomerular transections (Spearman r 0.833; p-value 0.010; data not shown). We could not find any further significant correlations comparing different amounts of glomerular transections (even between 150 and 300 transections) regarding the relative transcript expression of GAPDH and ADAMTS13 (Fig. 7b, c).

## Discussion

Both, RNA purity and quantity are decisive for successful mRNA expression studies. Our results confirm the previous finding, that the purity and quantity of RNA recovered from FFPE tissue are independent factors [25]. The variability of RNA purity and quantity may be due to different pre-analytical handling of the biopsy such as time to formalin fixation or rapid versus ordinary embedding [26, 27]. Moreover, different mRNA specimens seem to be affected differently by degradation [28]. Our goal was to document the RNA purity and quantity for quantitative mRNA analysis of micro-dissected glomerular compartments and to evaluate the approach according to the MIQE guidelines [29].

### RNA purity

Despite the certain degradation of our RNA samples, we had a similar range for RNA purity in between different samples as well as between different amounts of RNA, which seems to be valuable for further analyses [30]. As shown in Table 3, one recent study performed a comprehensive analysis for RNA purity, quality and quantity [9]. Beside this study no other documents the A260/A280 ratio, which might be due to the fact that it is difficult or even impossible to obtain reliable absorption values at A260 in micro-dissected FFPE tissue [31]. However, this does not necessarily mean that the RNA purity results are faulty. Landolt et al. have shown a mean A260/A280 ratio between 1.1 and 1.9 in 100 micro-dissected glomerular transections from FFPE biopsies [9], which was in the same range as in our samples. Most importantly, despite low A260/A280 ratios we could amplify mRNA transcripts, which demonstrates reasonable mRNA quality.

Another measure for RNA integrity is to calculate the ratio of 28S and 18S ribosomal RNA peak area [32]. However, it has recently been postulated that this manner of RNA integrity assessment from degraded FFPE samples is not a sensitive measure of RNA quality and samples with low RNA integrity numbers are still usable for RT-qPCR, provided that the amplicon length is sufficiently short (< 90 nucleotides) [33]. Therefore, we did not calculate RNA integrity.

Furthermore, studies have shown that the mean RNA fragment size—expressed as a DV_200_ value (percentage of RNA fragments > 200 nucleotides in size) and determined by a Fragment Analyzer (Advanced Analytical, Ankeny, USA) or Bioanalyzer (Agilent 2100, Agilent Technologies, Santa Clara, USA)—is a more reliable predictor for the successful RNA amplification of micro-dissected FFPE samples as compared to the conventional RNA integrity approach from regular tissue samples [9, 34–36]. In micro-dissected glomerular transections, there was a broad range for the DV_200_ values, from 26 to 89% in dependence of different RNA isolation methods [9]. However, the RNA quantity from micro-dissected glomerular transections is even below the pre-determined standard range of the Fragment Analyzer [9]. Therefore, due to the limited amount of RNA, we decided not to measure the DV_200_ value. Furthermore, in our opinion it is more important to insert all available micro-dissected RNA for reverse transcription and PCR itself. In case of 60 micro-dissected glomerular transections, we used a RNA concentration of 230 (182/277) ng/10 µl, which was a higher amount than requested for lowest RNA quality according to the stepwise approach of Landolt et al. [9]. In general, they suggest that if the DV_200_ is of high quality (> 70% of fragments > 200 nucleotides), a smaller amount of RNA (about 20 ng) is required; for medium quality (50–70%), an intermediate amount of RNA (about 20–40 ng); and for low quality (30–50%), a high amount of RNA (40–100 ng) is required [9].

### RNA quantity

In order to document RNA yields and transection amounts, we used two different approaches. (I) For *non*-*deparaffinized* glomerular transections, we conducted a comprehensive comparative study of different transection amounts, which revealed increasing but no proportional RNA concentrations. The total RNA isolated from 60 micro-dissected glomerular transections is higher than the range described in the study of Landolt et al. [9], which obtained a range of 138 ng (± 43 ng) RNA per 100 micro-dissected glomerular transections using the ExpressArt kit (Amsbio, Abingdon, UK) for RNA extraction. Independent of the RNA concentration, we detected no significant differences for the relative expression of ADAMTS13 and POLR2A between 20, 60 and 150 micro-dissected glomerular transections. For comparison, another non-renal study demonstrated similar mRNA (Epidermal growth factor receptor, Human epidermal growth factor receptor 2, Mouse double minute 2 homolog) expression levels within the range of 10^6^ to 100 micro-dissected cells in FFPE tissue [37]. Especially, strongly-expressed transcripts like POLR2A provide reproducible relative expression levels between 60 and 150 glomerular transections. Moreover, there was a lower inter-assay coefficient of variability for ADAMTS13, when at least 60 unstained glomerular transections were used. Samples with 20 micro-dissected glomerular transections revealed a non-detectable amplification signal for ADAMTS13 in four and for GAPDH in three out of eight analyzed cases. In another sample with 20 glomerular transections there was an off-scale C_q_ value for ADAMTS13 expression. Therefore, we conclude that 60 glomerular transections with a median RNA concentration of [23 (18/28) ng/µl] are necessary and sufficient for mRNA expression analysis. This number of transections can be reasonably achieved in most routine biopsies.

(II) For the amount of *deparaffinized and hemalaun*-*stained* Bowman’s capsule transections, we can only provide a rough estimate, because we performed no comprehensive analysis for different amounts of Bowman’s capsule transections. In all samples the RNA concentration was higher than the detection limit of spectrophotometers (10 ng/µl) [10]. About 300 hemalaun-stained micro-dissected Bowman’s capsule transections even revealed a higher amount of RNA compared to the mean concentration of 60 unstained glomerular transections. Therefore, we recommend micro-dissecting at least 300 hemalaun-stained Bowman’s capsule transections from FFPE renal biopsies. Interestingly, individual samples of our cohort with 180 Bowman’s capsule transections displayed higher RNA concentrations compared to 360 and 450 transections. Although samples were treated according to our diagnostic standard operation procedure, this unusual progression might be due to differences in pre-analytical steps, like the time period between harvesting and fixation of the tissue in formalin, which is crucial since any delay reduces the RNA quality and quantity [27].

In general, one has to keep in mind that concentration estimation can also be affected by phenol contamination, given that phenol absorbs strongly at 230 nm and ~ 270 nm. Indeed, in many samples we had high absorbance at 230 nm (data not shown). Therefore, the A260/A230 ratio was strongly reduced in our samples. However, there is no consensus on the acceptable lower limit of this ratio and the most important factor is the amount of phenol that is transferred to the downstream reaction, rather than the absorbance ratio. It is important to remember that absorbance ratios also depend on RNA concentration, especially in case of laser micro-dissection when RNA is at very low concentrations. Vice versa, absorbance around 260 nm can affect RNA concentration. Therefore, spectrophotometric analysis (e.g. Synergy, used in our study) of FFPE tissue yield different, consistently higher RNA concentrations compared to fluorometric analysis (e.g. Qubit) [38].

Furthermore, different RNA extraction methods yield different amounts of RNA [9]. We used the phenol–chloroform extraction because in case of highly-degraded RNA like FFPE autopsy tissue the phenol–chloroform extraction is still a valid alternative [39]. However, in whole non-renal tissue analysis, the High Pure kit (Roche, Mannheim, Germany) performed better than the conventional phenol–chloroform extraction method [40]. In micro-dissected glomerular FFPE transections, the High Pure kit and ExpressArt kit (Amsbio, Abingdon, UK) recovered the largest amounts of RNA [9]. Unfortunately, phenol–chloroform extraction was not used in their study [9]. Until a study compares the High Pure kit or ExpressArt kit to the phenol–chloroform extraction method in micro-dissected glomerular FFPE transections, both methods can be considered adequate.

### Operational issues for small amounts of starting material

To date, studies on PECs are limited because the small amounts of PECs could not be analyzed by quantitative real-time PCR for mRNAs. Our approach for laser micro-dissection can overcome this limitation. In contrast to all other studies shown in Table 3, we used the method of cDNA amplification. Especially for studying more than two targets and additional reference transcripts, we suggest increasing the amount of cDNA through this preliminary cDNA amplification step, which does not significantly distort relative mRNA levels [14, 17]. It has been shown that the combination of random primer-based cDNA synthesis and primer-specific preamplification provide best results [17]. The proposed primer-specific cDNA synthesis is not an option in our samples [41]. Furthermore, the variability of the preamplification—introduced into the experimental workflow of reverse transcription quantitative qPCR– is lower than the variability caused by the reverse transcription step [42]. Moreover, the amount of preamplified transcripts correlates with the initial cDNA target copy numbers in both good quality [43] and poor quality samples [44]. In order to avoid a bias, appropriate controls have to be used, like defined amounts of cell culture RNA, no-reverse-transcription controls and no-template controls.

Another aspect in case of micro-dissected glomerular transections with small amounts of starting material is how to manage reactions that do not give rise to any C_q_ value (missing C_q_ values). In order to avoid missing any low-level expressed transcripts, we suggest the pragmatic approach of replacing all off-scale C_q_ values (C_q_ > 35) and missing C_q_ values by C_q_ = 36 [19].

### Expression analysis in a single glomerulus

For certain projects, it could be relevant to compare gene expression levels between a normal (or less injured) glomerulus and a sclerotic glomerulus in the same biopsy sample. The diameter of a human glomerulus is on average about 150 µm. Therefore, in theory about 75 transections (cut at a thickness of 2 µm) could be cut from one single glomerulus. Indeed, this number of transections could provide sufficient RNA. However, in practice it will be difficult to identify a single glomerulus over multiple section levels. Therefore, it would be an approach to pool glomeruli with normal (or less injured) morphology as well as sclerotic glomeruli. For example, we would suggest micro-dissecting about 20 transections and pooling three glomeruli per biopsy.

### Reference transcripts

Twelve different candidate reference transcripts were tested for expression stability. All candidate reference transcripts are located on different chromosomes and are involved in different basic cellular processes, as shown in Table 2. This is a necessary requirement for reference transcripts [45]. We suggest using the geometric mean of PGK1 and PPIA as normalization factor in unstained glomerular transections. PPIA was also suggested as a reference transcript for the comparison of malignant and non-malignant kidney specimens [45, 46]. In contrast to our study Schmid et al. have shown that PPIA was expressed at low levels in glomeruli and thus it was claimed not to be suitable as a reference transcript for glomerular analysis [15]. One has to keep in mind that biopsies stored in RNA later were used in their study for micro-dissection. Furthermore, they demonstrated that gene expression studies of human kidney biopsies published from 1999 to 2002 predominantly used GAPDH as a reference transcript for RT-PCR studies [15]. In our study GAPDH does not belong to the most stable glomerular transcripts. GAPDH, PPIA and PGK1 were also recommended as reference transcripts in studies about mice with cystic kidney disease [47]. ACTB—used in some glomerular studies [12, 13, 48]—is no longer suggested as a reference transcript for RT-PCR in general [49]. This is confirmed by our results of less stably ACTB. Eukaryotic 18S rRNA is used as reference transcript in one other renal study [11], although this transcript is less stably expressed in our cohort. HPRT1, RPLPO [41] and B2M [50] have been described as renal reference transcripts and were detected in renal tumors. Here, we describe for the first time that HPRT1 and GUSB belong to the top four glomerular reference transcripts in human non-neoplastic kidney biopsies. However, RPLPO and B2M are less suitable. GUSB has also been described as a suitable reference gene in metastatic renal cell carcinoma [51].

### Limitations of the study

As discussed in Cohen et al. [10], a considerable variability of relative mRNA expression can be observed between consecutive sections. This variation could be a reason (I) for the decreasing inter-assay variability of relative ADAMTS13 expression with an increasing number of isolated glomerular transections and (II) for significant different expression levels of ADAMTS13, POLR2A and GAPDH in cases with 300 compared to 150 (ADAMTS13, POLR2A) or 60 (GAPDH) micro-dissected glomerular transections. This bias is negligible when relative expression values of different mRNA transcripts are compared in one defined amount of transections.

Furthermore, the number of biopsies for analyzing the effect of input RNA (patient cohort II) is difficult to increase due to the size limit of renal biopsies in clinical practice. However, even in our eight cases POLR2A revealed a reliable reproducibility for different amounts of RNA recovered from 60 to 150 micro-dissected glomerular transections when normalized to PGK1 and PPIA. There may be two reasons for the missing correlation of GAPDH and ADAMTS13 relative expression: (I) the small number of biopsies analyzed; or (II) the lower expression level of GAPDH and ADAMTS13 compared to POLR2A. Consequently, the inverse conclusion might be that fewer values of lower expressed transcripts have to be approached with caution.

Another aspect is the use of markers for discrimination between Bowman’s capsules and the tubulointerstitium. Due to the low amount of starting material, we focused on two positive markers being highly expressed in podocytes but being also expressed at lower level in Bowman’s capsules (WT1 and GLEPP1). However, these markers are not expressed in the tubulointerstitium. To the best of our knowledge, it is difficult to find negative markers for Bowman’s capsules, which could further exclude the contamination of tubular cells and interstitial cells in RNA from Bowman’s capsules.

Furthermore, due to the low amount of starting material we were not able to perform a comprehensive reference gene analysis in hemalaun-stained micro-dissected Bowman’s capsule transections. Therefore, we have chosen the geometric mean of PGK1 and GAPDH as normalization factor for PEC, tubulointerstitium and glomeruli on the basis of the current literature [23, 24].

## Conclusion

In summary, we show the feasibility for micro-dissecting glomerular and Bowman’s capsule transections followed by evidence-based recommendations for quantitative mRNA analysis. We recommend micro-dissecting at least 60 unstained glomerular or 300 hemalaun-stained Bowman’s capsule transections from FFPE renal biopsies to obtain reliable results equivalent to larger amounts of tissue. We provide evidence that RNA quantity is within an acceptable range even for low RNA purity. Therefore, we suggest the following pragmatic approach: in case of 60 available unstained glomerular transections, quantity and purity control is optional for documentation but not gainful regarding further analytical steps, because (I) the range of RNA concentrations in equal amounts of micro-dissected glomerular transections is low and (II) appropriate normalization of C_q_ values using our suggested reference transcripts (PGK1 and PPIA) can compensate for different amounts of RNA purity and quantity [52]. However, with fewer than 60 available unstained glomerular transections either quality and quantity control should be performed (e.g. measuring the DV_200_ value and fluorometric analysis) or the sample has to be excluded.

## Additional file

**Additional file 1.** Additional tables.

## Abbreviations

ACTB: Beta-Aktin
ADAMTS13: a disintegrin and metalloproteinase with thrombospondin type 1 motives 13
ATN: acute tubular injury
B2M: beta-2 microglobulin
cDNA: complementary DNA
C_q_: quantification cycle
DAB: diaminobenzidine
DEPC: diethyl pyrocarbonate
DNA: deoxyribonucleic acid
DV_200_: percentage of RNA fragments > 200 nucleotides in size
FFPE: formalin-fixed paraffin-embedded
GAPDH: glyceraldehyde-3-phosphate dehydrogenase
GUSB: glucuronidase, beta
HMBS: hydroxymethylbilane synthase
HPRT1: hypoxanthine phosphoribosyltransferase 1
LCM: laser capture micro-dissection
MIQE: Minimum Information for Publication of Quantitative Real-Time PCR Experiments
MMI^®^: Molecular Machines and Industries
mRNA: messenger RNA
PAX2: paired box 2
PCR: polymerase chain reaction
PECs: parietal epithelial cells
PGK1: phosphoglycerate kinase 1
POLR2A: RNA polymerase II subunit A
PPIA: peptidylprolyl isomerase A
PTPRO (alias GLEPP1): protein tyrosine phosphatase, receptor type O, alias GLEPP1
RNA: ribonucleic acid
RPLPO: ribosomal protein lateral stalk subunit P0
rRNA: ribosomal RNA
RT-qPCR: reverse-transcription quantitative PCR
TBP: TATA-box binding protein
V: average pairwise variation
WT1: wilms tumor 1
