# Supplementary material for: Recommendations for mRNA analysis of micro-dissected glomerular tufts from paraffin-embedded human kidney biopsy samples
Source: BMC Mol Biol. 2018 Mar 13;19:2. doi: 10.1186/s12867-018-0103-x (PMC5850911; doi:10.1186/s12867-018-0103-x)
Supplement: Supplementary file 1 — Additional file 1. Additional tables. [file 12867_2018_103_MOESM1_ESM.docx]

**Additional information file for all generated and analysed data. Data are given in the order of appearance in the article.**

**Table S1: Additional data for table 1.**

|  | **Age at biopsy** | **Creatinine (mg/dl)** |
| --- | --- | --- |
| **Patient sample cohort I** | 71 | 1.8 |
|  | 53 | 1.4 |
|  | 55 | 1.2 |
|  | 24 | 5.6 |
|  | 37 | 3.1 |
|  | 71 | 1.7 |
|  | 16 | 5.0 |
|  | 26 | 3.5 |
|  | 15 | 5.6 |
|  |  |  |
| **Patient sample cohort II** | 30 | 0.7 |
|  | 37 | 1.5 |
|  | 46 | 0.8 |
|  | 61 | 2 |
|  | 72 | 1.4 |
|  | 73 | Dialysis |
|  | 9 | 0.3 |
|  | 74 | 0.8 |
|  | 57 | 0.77 |
|  | 26 | 9.1 |
|  | 44 | 2.7 |
|  | 55 | Dialysis |
|  | 50 | 1.6 |
|  | 55 | 4.7 |
|  | 48 | 3.5 |

**Table S2: Cq values and relative mRNA expression levels of selected transcripts in micro-dissected non-injured hemalaun-stained glomerular substructures (Additional data for Fig. 3).**

|  | **C_q_ values WT1** | | |
| --- | --- | --- | --- |
| **Sample#** | **Glomerular tuft** | **PECs** | **Periglom. tubulo-interstitium** |
| 1 | 18.85 | 24.01 | 28.25 |
| 2 | 15.34 | Not detectable | 37.01 |
| 3 | 16.57 | 20.45 | 24.30 |
| 4 | 19.85 | 22.66 | 25.31 |
| 5 | 18.21 | 22.14 | 29.39 |
| 6 | 18.25 | 22.21 | 30.17 |
| 7 | 19.57 | 25.42 | 28.57 |
| 8 | 21.55 | 31.52 | 30.05 |
| 9 | 15.30 | 23.33 | 28.34 |

| **Fig . 3A** | **Relative expression WT1** | | |
| --- | --- | --- | --- |
| **Sample#** | **Glomerular tuft** | **PECs** | **Periglom. tubulo-interstitium** |
| 1 | 40.48 | 3.61 | 0.04 |
| 2 | 5.87 | Not detectable | 0.00 |
| 3 | 6.68 | 5.20 | 0.02 |
| 4 | 14.82 | 3.33 | 0.08 |
| 5 | 17.03 | 2.94 | 0.01 |
| 6 | 7.41 | 1.15 | 0.00 |
| 7 | 4.06 | 1.91 | 0.03 |
| 8 | 8.78 | 0.96 | 0.03 |
| 9 | Outlier | 20.15 | 0.07 |

|  | **C_q_ values GLEPP-1** | | |
| --- | --- | --- | --- |
| **Sample#** | **Glomerular tuft** | **PECs** | **Periglom. tubulo-interstitium** |
| 1 | 19.91 | 26.77 | 28.44 |
| 2 | 14.87 | Not detectable | 38.37 |
| 3 | 19.93 | 23.30 | 17.13 |
| 4 | 21.76 | 24.95 | 27.31 |
| 5 | 19.76 | 23.05 | 29.47 |
| 6 | 18.91 | 23.73 | 30.17 |
| 7 | 20.04 | 26.49 | 28.57 |
| 8 | 23.40 | 33.06 | 31.64 |
| 9 | 15.99 | 31.31 | 30.82 |

| **Fig. 3B** | **Relative expression GLEPP-1** | | |
| --- | --- | --- | --- |
| **Sample#** | **Glomerular tuft** | **PECs** | **Periglom. tubulo-interstitium** |
| 1 | 19.34 | 0.53 | 0.03 |
| 2 | 8.16 | Not detectable | 0.00 |
| 3 | 0.65 | 0.72 | 2.87 |
| 4 | 3.95 | 0.68 | 0.02 |
| 5 | 5.83 | 1.57 | 0.01 |
| 6 | 4.68 | 0.40 | 0.00 |
| 7 | 2.92 | 0.91 | 0.03 |
| 8 | 2.42 | 0.33 | 0.01 |
| 9 | Outlier | 0.08 | 0.01 |

|  | **C_q_ values PAX2** | | |
| --- | --- | --- | --- |
| **Sample#** | **Glomerular tuft** | **PECs** | **Periglom. tubulo-interstitium** |
| 1 | 25.30 | 24.57 | 23.40 |
| 2 | 36.85 | Not detectable | 15.19 |
| 3 | 24.95 | 21.62 | 16.98 |
| 4 | 28.06 | 23.36 | 20.22 |
| 5 | 27.98 | 23.19 | 23.52 |
| 6 | 24.08 | 21.81 | 14.81 |
| 7 | 26.21 | 26.54 | 25.18 |
| 8 | 30.32 | 31.17 | 24.37 |
| 9 | 37.47 | 22.46 | 22.94 |

| **Fig. 3C** | **Relative expression PAX2** | | |
| --- | --- | --- | --- |
| **Sample#** | **Glomerular tuft** | **PECs** | **Periglom. tubulo-interstitium** |
| 1 | 0.46 | 2.44 | 1.05 |
| 2 | 0.00 | Not detectable | 3.42 |
| 3 | 0.02 | 2.30 | 3.19 |
| 4 | 0.05 | 2.04 | 2.73 |
| 5 | 0.02 | 1.42 | 0.41 |
| 6 | 0.13 | 1.51 | 4.20 |
| 7 | 0.04 | 0.88 | 0.27 |
| 8 | 0.02 | 1.23 | 1.54 |
| 9 | 0.00 | 36.82 | 2.83 |

|  | **C_q_ values GAPDH** | | |
| --- | --- | --- | --- |
| **Sample#** | **Glomerular tuft** | **PECs** | **Periglom. tubulo-interstitium** |
| 1 | 23.80 | 24.37 | 24.00 |
| 2 | 13.17 | 28.22 | 11.58 |
| 3 | 20.41 | 23.52 | 20.12 |
| 4 | 25.35 | 25.61 | 23.33 |
| 5 | 24.67 | 24.37 | 23.56 |
| 6 | 22.01 | 23.39 | 16.77 |
| 7 | 23.56 | 27.10 | 24.88 |
| 8 | 25.93 | 27.50 | 26.26 |
| 9 | 23.86 | 28.29 | 26.76 |

|  | **C_q_ values PGK1** | | |
| --- | --- | --- | --- |
| **Sample#** | **Glomerular tuft** | **PECs** | **Periglom. tubulo-interstitium** |
| 1 | 24.58 | 27.44 | 22.93 |
| 2 | 24.32 | Not detectable | 24.86 |
| 3 | 18.27 | 22.15 | 17.29 |
| 4 | 22.23 | 23.23 | 20.12 |
| 5 | 20.16 | 23.04 | 21.01 |
| 6 | 20.30 | 21.47 | 16.99 |
| 7 | 19.78 | 25.63 | 21.80 |
| 8 | 23.49 | 36.00 | 23.79 |
| 9 | 23.85 | 27.05 | 22.33 |

**Table S3: RNA concentrations in hemalaun-stained micro-dissected glomerular substructures (Additional data for Fig. 4).**

|  |  | **RNA(ng/µl)** | | |
| --- | --- | --- | --- | --- |
| **Sample#** | **Number of microdissected**  **glomerular tuft transections** | | **Glomerular tufts** | **Bowman's capsule transections** |
| 1 | 67 | | 6.4 | 10.8 |
| 2 | 27 | | 11.7 | 10.5 |
| 3 | 65 | | 25.8 | 4.4 |
| 4 | 418 | | 33.7 | 57.2 |
| 5 | 517 | | 42.4 | 56.2 |
| 6 | 335 | | 50.0 | 31.7 |
| 7 | 463 | | 70.8 | 43.8 |
| 8 | 195 | | 124.8 | 109.3 |
| 9 | 504 | | 166.2 | 119.9 |

**Table S4: C_q_ values of selected reference transcripts. These C_q_ values were used for the NormFinder and geNormPlus approach (except sample 15, which was excluded due to a not detectable (n.d.) sample for Hmbs).**

| **Sample** | **18 S** | **beta2Mikroglobulin** | **GAPDH** | **GUSB** | **Hmbs** | **HPRT** | **PGK1** | **POLR2A** | **PPIA** | **RPLPO** | **TBP** | **Aktin** |
| --- | --- | --- | --- | --- | --- | --- | --- | --- | --- | --- | --- | --- |
| 1 | 6.57 | 16.90 | 26.40 | 25.50 | 26.58 | 26.88 | 23.57 | 22.83 | 22.84 | 22.22 | 26.31 | 18.66 |
| 2 | 8.17 | 18.87 | 27.83 | 27.51 | 36.69 | 28.86 | 25.59 | 24.06 | 24.63 | 24.89 | 29.48 | 20.09 |
| 3 | 6.23 | 17.35 | 27.07 | 24.83 | 28.15 | 26.93 | 24.02 | 23.04 | 22.79 | 21.76 | 27.71 | 18.31 |
| 4 | 7.25 | 17.86 | 26.71 | 25.71 | 38.74 | 28.45 | 24.71 | 24.55 | 24.11 | 23.92 | 25.92 | 19.42 |
| 5 | 7.23 | 16.68 | 28.43 | 26.31 | 27.98 | 26.69 | 24.56 | 22.95 | 23.91 | 22.59 | 26.75 | 18.39 |
| 6 | 5.77 | 15.28 | 25.53 | 24.85 | 26.11 | 25.45 | 23.92 | 22.27 | 22.21 | 21.77 | 25.72 | 17.71 |
| 7 | 4.76 | 15.87 | 24.23 | 23.69 | 25.90 | 24.95 | 22.23 | 22.11 | 21.32 | 20.24 | 24.67 | 18.42 |
| 8 | 5.17 | 14.76 | 24.30 | 23.50 | 25.49 | 24.78 | 21.89 | 22.00 | 21.65 | 20.36 | 25.27 | 16.89 |
| 9 | 5.58 | 14.34 | 24.86 | 23.60 | 27.67 | 24.30 | 21.92 | 21.11 | 21.06 | 20.11 | 24.52 | 16.26 |
| 10 | 5.90 | 16.52 | 26.16 | 24.57 | 27.89 | 25.84 | 23.21 | 21.50 | 22.06 | 21.68 | 25.84 | 17.29 |
| 11 | 5.25 | 16.05 | 24.90 | 24.50 | 27.25 | 24.73 | 22.70 | 21.96 | 21.35 | 21.19 | 25.19 | 18.56 |
| 12 | 6.71 | 18.28 | 27.93 | 27.02 | 33.48 | 28.22 | 25.77 | 24.79 | 24.78 | 22.59 | 26.59 | 21.53 |
| 13 | 7.82 | 19.72 | 28.62 | 27.29 | 41.12 | 28.50 | 25.36 | 24.47 | 24.67 | 23.49 | 28.80 | 20.63 |
| 14 | 5.49 | 15.54 | 24.85 | 23.69 | 26.07 | 24.74 | 22.30 | 21.16 | 21.26 | 20.47 | 25.23 | 17.25 |
| 15 | 7.27 | 18.18 | 29.82 | 27.37 | N.d. | 28.11 | 24.75 | 23.23 | 23.90 | 17.00 | 26.22 | 19.01 |

**Table S5: Stability of selected reference transcripts according to the algorithms of geNormPlus and NormFinder (Additional data for Fig. 5).**

| **Candidate reference** | **NormFinder** |  | **Candidate reference** | **geNormPlus** |  | **Ratio of average pairwise variation** | |
| --- | --- | --- | --- | --- | --- | --- | --- |
| HPRT1 | 0.0100 |  | PGK 1 | 0.3710 |  | V2/3 | 0.115 |
| PPIA | 0.0110 |  | PPIA | 0.3770 |  | V3/4 | 0.131 |
| PGK1 | 0.0120 |  | GUSB | 0.3810 |  | V4/5 | 0.103 |
| GUSB | 0.0120 |  | HPRT1 | 0.4730 |  | V5/6 | 0.092 |
| GAPDH | 0.0140 |  | POLR2A | 0.5200 |  | V6/7 | 0.08 |
| POLR2A | 0.0150 |  | RPLPO | 0.5610 |  | V7/8 | 0.078 |
| RPLPO | 0.0150 |  | 18S rRNA | 0.5900 |  | V8/9 | 0.071 |
| TBP | 0.0180 |  | B2m | 0.6260 |  | V9/10 | 0.075 |
| B2m | 0.0220 |  | GAPDH | 0.6540 |  | V10l/11 | 0.073 |
| BACT | 0.0260 |  | BACT | 0.6950 |  | V11/12 | 0.351 |
| 18S rRNA | 0.0420 |  | TBP | 0.7370 |  |  |  |
| HMBS | 0.0450 |  | HMBS | 1.3210 |  |  |  |

**Table S6: Comparing different unstained glomerular transection counts in eight cases of cohort II (Additional data for Fig. 6 and 7).**

| Fig. 6A | **Total extracted RNA (ng/µl)** | | | |
| --- | --- | --- | --- | --- |
| Sample# | 20 Glomeruli | 60 Glomeruli | 150 Glomeruli | 300 Glomeruli |
| 1 | 12.3 | 15.7 | 40.2 | 74.4 |
| 2 | 19.2 | 25.7 | 47.6 | 90.2 |
| 3 | 17.4 | 20.3 | 52.5 | 70.4 |
| 4 | 8.5 | 26.7 | 61.1 | 105.0 |
| 5 | 27.2 | 18.7 | 64.9 | 51.5 |
| 6 | 15.5 | 28.0 | 33.1 | 27.5 |
| 7 | 25.7 | 18.0 | 26.1 | 60.0 |
| 8 | 16.0 | 28.2 | 46.3 | 64.3 |

| Fig. 6B | **RNA A260/A280 ratio** | | | |
| --- | --- | --- | --- | --- |
| Sample# | 20 Glomeruli | 60 Glomeruli | 150 Glomeruli | 300 Glomeruli |
| 1 | 1.40 | 1.50 | 1.89 | 2.04 |
| 2 | 1.18 | 1.31 | 1.66 | 1.95 |
| 3 | 1.24 | 1.43 | 1.64 | 1.80 |
| 4 | 0.89 | 1.79 | 1.92 | 2.02 |
| 5 | 2.27 | 1.40 | 1.78 | 1.63 |
| 6 | 1.64 | 1.50 | 1.74 | 1.55 |
| 7 | 1.74 | 1.49 | 1.17 | 1.51 |
| 8 | 1.34 | 1.76 | 1.71 | 1.85 |

|  | **C_q_ values ADAMTS13** | | | |
| --- | --- | --- | --- | --- |
| Sample# | 20 Glomeruli | 60 Glomeruli | 150 Glomeruli | 300 Glomeruli |
| 1 | 36.00 | 29.18 | 36.00 | 26.33 |
| 2 | 36.00 | 30.55 | 27.27 | 27.29 |
| 3 | 36.00 | 26.27 | 26.29 | 26.96 |
| 4 | 29.98 | 26.27 | 27.04 | 26.65 |
| 5 | 26.36 | 28.72 | 26.70 | 23.97 |
| 6 | 36.00 | 36.42 | 27.33 | 24.63 |
| 7 | 36.00 | 34.78 | 26.98 | 24.92 |
| 8 | 27.32 | 26.00 | 24.20 | 24.44 |

| Fig. 6C | **Relative expression of ADAMTS13** | | | |
| --- | --- | --- | --- | --- |
| Sample# | 20 Glomeruli | 60 Glomeruli | 150 Glomeruli | 300 Glomeruli |
| 1 | 0.00 | 0.01 | 0.00 | 0.04 |
| 2 | 0.00 | 0.03 | 0.06 | 0.08 |
| 3 | 0.00 | 0.05 | 0.02 | 0.01 |
| 4 | 0.01 | 0.04 | 0.01 | 0.01 |
| 5 | 0.17 | 0.02 | 0.02 | 0.04 |
| 6 | 0.00 | 0.00 | 0.03 | 0.05 |
| 7 | 0.00 | 0.00 | 0.02 | 0.04 |
| 8 | 0.05 | 0.05 | 0.03 | 0.03 |

| Fig. 6D | **Inter-assay coefficient of variability for expression of ADAMTS13** |
| --- | --- |
| 20 vs. 60 Glomeruli | 149.41 |
| 20 vs. 150 Glomeruli | 143.94 |
| 20 vs. 300 Glomeruli | 133.1 |
| 60 vs. 150 Glomeruli | 86.53 |
| 60 vs. 300 Glomeruli | 75.69 |
| 150 vs. 300 Glomeruli | 70.22 |

|  | **C_q_ values GAPDH** | | | |
| --- | --- | --- | --- | --- |
| Sample# | 20 Glomeruli | 60 Glomeruli | 150 Glomeruli | 300 Glomeruli |
| 1 | 36.00 | 24.23 | 25.64 | 23.81 |
| 2 | 36.00 | 27.93 | 25.89 | 26.75 |
| 3 | 26.85 | 24.90 | 23.58 | 22.59 |
| 4 | 24.31 | 24.86 | 22.64 | 22.34 |
| 5 | 35.92 | 26.16 | 25.03 | 22.23 |
| 6 | 36.00 | 29.82 | 24.84 | 23.56 |
| 7 | 27.85 | 28.62 | 24.16 | 22.93 |
| 8 | 25.33 | 24.85 | 21.41 | 21.77 |

| Fig. 6E | **Relative expression of GAPDH** | | | |
| --- | --- | --- | --- | --- |
| Sample# | 20 Glomeruli | 60 Glomeruli | 150 Glomeruli | 300 Glomeruli |
| 1 | 0.00 | 0.18 | 0.09 | 0.25 |
| 2 | 0.00 | 0.16 | 0.15 | 0.11 |
| 3 | 0.16 | 0.14 | 0.13 | 0.24 |
| 4 | 0.23 | 0.10 | 0.11 | 0.13 |
| 5 | 0.00 | 0.09 | 0.06 | 0.12 |
| 6 | 0.00 | 0.02 | 0.15 | 0.10 |
| 7 | 0.11 | 0.08 | 0.11 | 0.15 |
| 8 | 0.21 | 0.12 | 0.20 | 0.20 |

|  | **C_q_ values POLR2A** | | | |
| --- | --- | --- | --- | --- |
| Sample# | 20 Glomeruli | 60 Glomeruli | 150 Glomeruli | 300 Glomeruli |
| 1 | 25.96 | 22.11 | 21.99 | 21.19 |
| 2 | 25.68 | 24.79 | 22.80 | 22.60 |
| 3 | 24.22 | 21.96 | 20.63 | 20.37 |
| 4 | 21.92 | 21.11 | 19.38 | 19.14 |
| 5 | 22.93 | 21.50 | 20.40 | 18.58 |
| 6 | 27.58 | 23.23 | 21.57 | 19.51 |
| 7 | 24.90 | 24.47 | 20.96 | 20.24 |
| 8 | 22.34 | 21.16 | 18.80 | 19.23 |

| Fig. 6F | **Relative expression of POLR2A** | | | |
| --- | --- | --- | --- | --- |
| Sample# | 20 Glomeruli | 60 Glomeruli | 150 Glomeruli | 300 Glomeruli |
| 1 | 1.31 | 0.79 | 1.15 | 1.52 |
| 2 | 1.26 | 1.40 | 1.26 | 1.95 |
| 3 | 1.01 | 1.04 | 1.03 | 1.14 |
| 4 | 1.21 | 1.30 | 1.04 | 1.17 |
| 5 | 1.83 | 2.19 | 1.40 | 1.54 |
| 6 | 0.86 | 2.13 | 1.44 | 1.68 |
| 7 | 0.85 | 1.46 | 1.04 | 0.96 |
| 8 | 1.64 | 1.53 | 1.23 | 1.18 |

|  | **C_q_ values PGK1** | | | |
| --- | --- | --- | --- | --- |
| Sample# | 20 Glomeruli | 60 Glomeruli | 150 Glomeruli | 300 Glomeruli |
| 1 | 27.18 | 22.23 | 22.93 | 22.27 |
| 2 | 26.39 | 25.77 | 23.94 | 24.53 |
| 3 | 25.23 | 22.70 | 21.35 | 21.10 |
| 4 | 22.52 | 21.92 | 19.98 | 19.88 |
| 5 | 24.76 | 23.21 | 21.37 | 19.75 |
| 6 | 28.03 | 24.75 | 22.44 | 20.69 |
| 7 | 24.95 | 25.36 | 21.51 | 20.74 |
| 8 | 23.34 | 22.30 | 19.57 | 19.96 |

|  | **C_q_ values PPIA** | | | |
| --- | --- | --- | --- | --- |
| Sample# | 20 Glomeruli | 60 Glomeruli | 150 Glomeruli | 300 Glomeruli |
| 1 | 25.55 | 21.32 | 21.48 | 21.33 |
| 2 | 25.64 | 24.78 | 22.36 | 22.65 |
| 3 | 23.27 | 21.35 | 20.01 | 20.03 |
| 4 | 21.88 | 21.06 | 18.90 | 18.87 |
| 5 | 22.87 | 22.06 | 20.41 | 18.66 |
| 6 | 26.69 | 23.90 | 21.76 | 19.82 |
| 7 | 24.40 | 24.67 | 20.55 | 19.65 |
| 8 | 22.75 | 21.26 | 18.64 | 18.98 |
